# Crystal structure of 1,13,14-tri­aza­dibenz[*a*,*j*]anthracene 1,1,2,2-tetra­chloro­ethane monosolvate

**DOI:** 10.1107/S2056989015009263

**Published:** 2015-05-23

**Authors:** Take-aki Koizumi, Tomohiro Hariu, Yoshihisa Sei

**Affiliations:** a Chemical Resources Laboratory, Tokyo Institute of Technology, 4259 nagatsuta, Midori-ku, Yokohama 226-8503, Japan

**Keywords:** crystal structure, 1,9,10-anthyridine, hydrogen bonding, π–π stacking

## Abstract

The asymmetric unit of the title compound, C_19_H_11_N_3_·C_2_H_2_Cl_4_, consists of one half-mol­ecule of 1,13,14-tri­aza­dibenz[*a*,*j*]anthracene (dibenzo[*c*,*h*]-1.9,10-anthyridine, dbanth) and one half of 1,1,2,2-tetra­chloro­ethane (TCE), both of which are located on a crystallographic twofold rotation axis. The dihedral angle between the planes of the terminal benzene rings in dbanth is 3.59 (7)° owing to the steric repulsion between the H atoms in the two benzo groups and the H atom in the central pyridine ring of the anthridine skeleton. In the crystal, π–π inter­actions between pyridine rings [centroid–centroid distances = 3.568 (2) and 3.594 (2) Å] link the dbanth mol­ecules to form a one-dimensional columnar structure along the *c* axis. The dbanth and TCE mol­ecules are connected through weak bifurcated C—H⋯(N,N) hydrogen bonds.

## Abstract 2

The crystal structure of 1,1,2,2-tetra­chloro­ethane (TCE)-solvated 1,13,14-tri­aza­dibenz[*a*,*j*]anthracene (dibenzo[*c*,*h*]-1.9,10-anthyridine, dbanth) was determined by X-ray diffraction study. Two H atoms in the solvated TCE mol­ecule form inter­molecular C—H⋯(N,N) hydrogen bonds with three N atoms in dbanth. π–π inter­actions link the dbanth mol­ecules to form a one-dimensional columnar structure.

## Chemical context   

1,9,10-Anthyridine has an anthracene skeleton with three imine N atoms that are situated at the same edge of the mol­ecule. Since an imine unit in an aromatic compound such as pyridine can act as a hydrogen-bond acceptor, 1,9,10-anthyridine can form a triply hydrogen-bonded structure with a corresponding H-atom donor, such as 2,6-di­amino­pyri­dinium and 2,6-bis­(hy­droxy­meth­yl)phenol (Murray & Zimmerman, 1992 ▸; Xu *et al.*, 2006 ▸; Djurdjevic *et al.*, 2007 ▸; Blight *et al.*, 2009 ▸). Formation of multiple hydrogen bonds often corresponds to a large association constant (*K*_a_ = *ca* 10^4^–10^10^); therefore, 1,9,10-anthyridine derivatives are promising components for supra­molecular compounds. However, there have been few reports on the crystal structures of 1,9,10-anthyridine derivatives. The crystal structure and inter­molecular inter­actions of chloro­benzene-solvated 2,3,7,8-tetra­phenyl-1,9,10-anthyridine have been reported (Madhavi *et al.*, 1997 ▸). In addition, 1,13,14-tri­aza­dibenz[*a*,*j*]anthracene (dbanth) has been synthesized and its crystal structure has been reported (Djurdjevic *et al.*, 2007 ▸; Blight *et al.*, 2009 ▸). In that case, the crystals contained no solvent mol­ecules. In other instances, several transition-metal complexes bearing dbanth as a ligand have been reported (Wang *et al.*, 2012 ▸; Huang *et al.*, 2013 ▸; Hirakawa & Koizumi, 2014 ▸). In this paper, we report the crystal structure of dbanth 1,1,2,2-tetra­chloro­ethane (TCE) monosolvate, (I). The H atoms in the TCE mol­ecule form C—H⋯N hydrogen bonds with three dbanth N atoms (Table 1 ▸). 
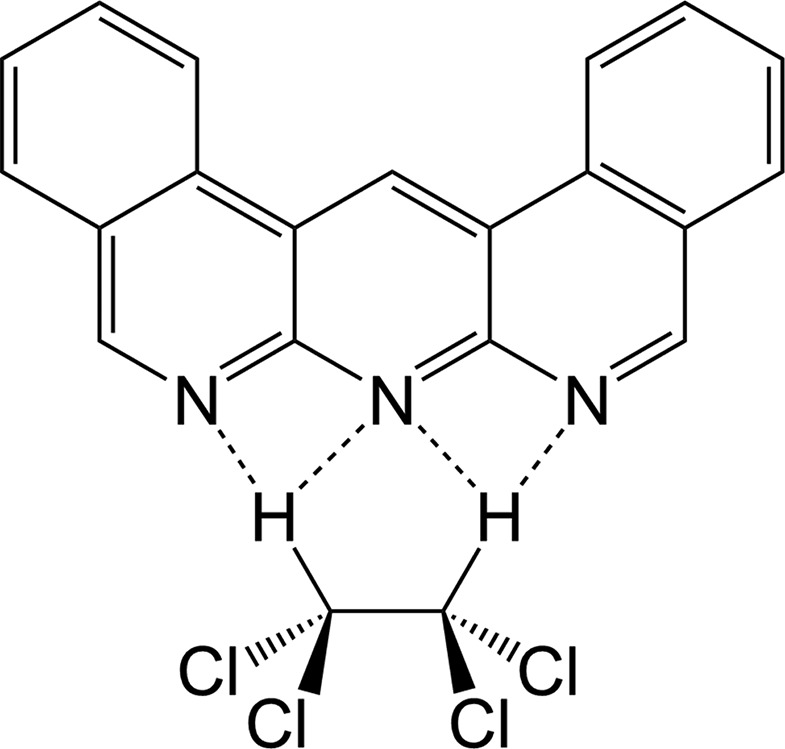


## Structural commentary   

**Figure 1:**
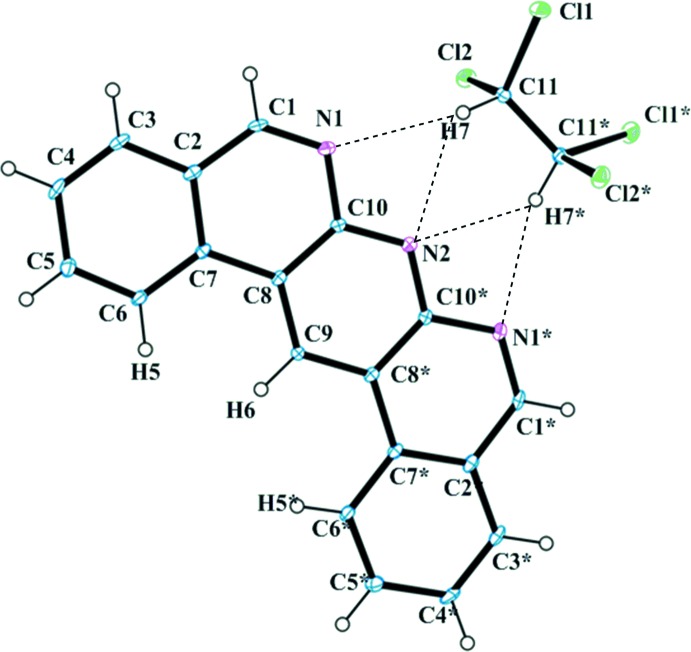
The two components of the title compound, (I), with displacement ellipsoids drawn at the 50% probability level. C—H⋯N hydrogen bonds are shown as dashed lines. [Symmetry code: (*) −*x* + 1, *y*, −*z* + 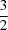.]

The mol­ecular structure of the title compound is depicted in Fig. 1 ▸. The dbanth and TCE mol­ecules have twofold rotation symmetry. Although the structure of dbanth is almost planar, the planes of the terminal benzene rings are slightly twisted with respect to each other, with a dihedral angle of 3.59 (7)°. The distortion of the compound is considered to be due to the steric repulsion between atoms H5, H5* and H6. Atom H7 in the solvated TCE mol­ecule forms a bifurcated hydrogen bond with the two N atoms (N1 and N2) of the dbanth mol­ecule (Table 1 ▸). When dbanth was recrystallized from CHCl_3_, solvation of CHCl_3_ did not occur. This result indicates that formation of C—H⋯N hydrogen bonds stabilizes the 1:1 complex of dbanth and TCE.

## Supra­molecular features   

**Figure 2:**
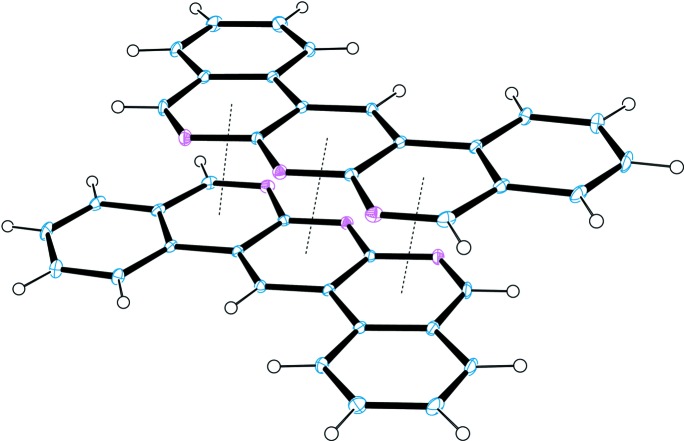
A partial packing diagram of the title compound, showing π–π inter­actions (dotted lines).

**Figure 3:**
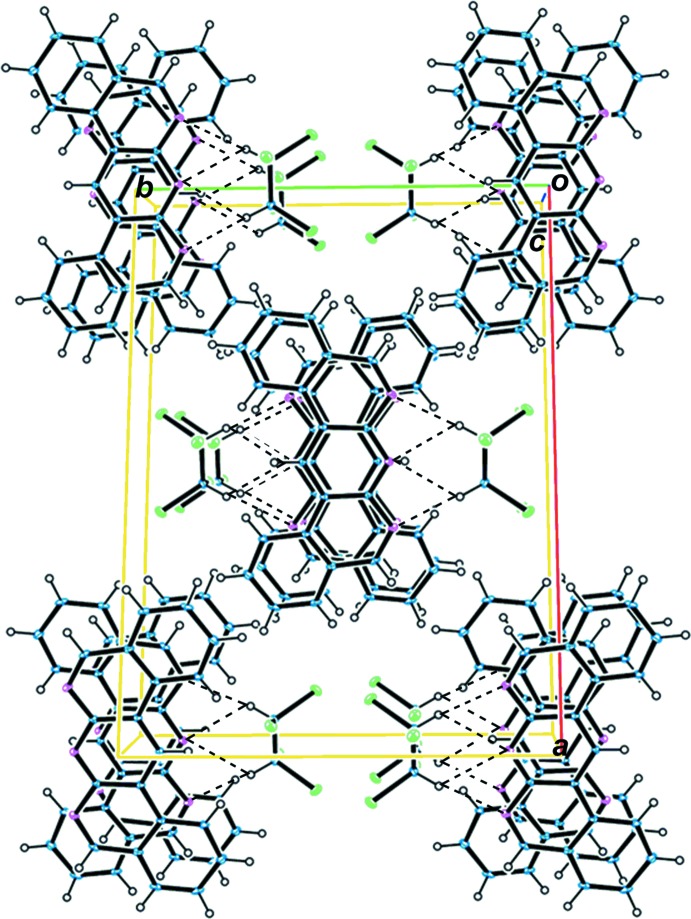
A crystal packing of the title compound, viewed down the *c* axis. Dashed lines indicate C—H⋯N hydrogen bonds.

In the crystal, the dbanth mol­ecule inter­acts with the neighbouring dbanth mol­ecule through π–π stacking inter­actions, with an average inter­planar distance of 3.36 Å; the centroid–centroid distances between pyridine rings containing atom N1 and between pyridine rings containing atom N2 are 3.568 (2) and 3.594 (2) Å, respectively (Fig. 2 ▸). The dbanth mol­ecules form a one-dimensional columnar structure *via* successive π–π stacking inter­actions (Fig. 3 ▸). A twofold rotation axis passes through atoms N2, C9 and H6 of the central pyridine ring, so that all of the dbanth mol­ecules are arranged parallel to one another in the space group *C*2/*c.* In the crystal of nonsolvated dbanth (space group *P*2_1_/*c*; Djurdjevic *et al.*, 2007 ▸), dbanth mol­ecules are also stacked in a column, but the mol­ecules in the neighbouring columns are inclined to each other by 41.8 (2)°.

## Synthesis and crystallization   

1,13,14-Tri­aza­dibenz[*a*,*j*]anthracene (dbanth) was synthesized *via* the reaction of 2,6-di­amino-3,5-di­iodo­pyridine with two equivalents of 2-formyl­benzene­boronic acid using Pd(PPh_3_)_4_ as a catalyst according to a literature method (Djurdjevic *et al.*, 2007 ▸). Single crystals suitable for X-ray diffraction were obtained from a TCE solution by slow evaporation.

## Refinement   

Crystal data, data collection, and refinement details are summarized in Table 2 ▸. All H atoms were fixed geometry (C—H = 0.93 or 0.98 Å) and refined using a riding model, with *U*_iso_(H) values set at 1.2*U*_eq_ of the parent atom.

## Supplementary Material

Crystal structure: contains datablock(s) General, I. DOI: 10.1107/S2056989015009263/is5393sup1.cif

Structure factors: contains datablock(s) I. DOI: 10.1107/S2056989015009263/is5393Isup2.hkl

Supporting information file. DOI: 10.1107/S2056989015009263/is5393Isup3.cml

CCDC references: 1401209, 1401209

Additional supporting information:  crystallographic information; 3D view; checkCIF report

## Figures and Tables

**Table 1:** Hydrogen-bond geometry (, )

*D*H*A*	*D*H	H*A*	*D* *A*	*D*H*A*
N1H7C11	0.98	2.53	3.372(3)	144
N2H7C11	0.98	2.57	3.206(3)	122

**Table 2:** Experimental details

Crystal data	
Chemical formula	C_19_H_11_N_3_C_2_H_2_Cl_4_
*M*_r_	449.14
Crystal system, space group	Monoclinic, *C*2/*c*
Temperature (K)	90
*a*, *b*, *c* ()	20.072(7), 14.190(5), 7.079(3)
()	110.255(4)
*V* (^3^)	1891.5(11)
*Z*	4
Radiation type	Mo *K*
(mm^1^)	0.64
Crystal size (mm)	0.79 0.40 0.10

Data collection	
Diffractometer	Bruker APEXII CCD area detector
Absorption correction	Multi-scan (*SADABS*; Bruker, 1996 ▸)
*T*_min_, *T*_max_	0.511, 0.938
No. of measured, independent and observed [*F* ^2^ > 2(*F* ^2^)] reflections	4336, 1670, 1606
*R*_int_	0.038
(sin /)_max_ (^1^)	0.595

Refinement	
*R*[*F* ^2^ > 2(*F* ^2^)], *wR*(*F* ^2^), *S*	0.030, 0.081, 1.06
No. of reflections	1670
No. of parameters	128
H-atom treatment	H-atom parameters constrained
_max_, _min_ (e ^3^)	0.50, 0.27

Computer programs: *APEX2* (Bruker, 2006 ▸), *SAINT* (Bruker, 2004 ▸), *SHELXT* (Sheldrick, 2015*a* ▸), *SHELXL2014* (Sheldrick, 2015*b* ▸) and *CrystalStructure* (Rigaku, 2010 ▸).

## supplementary crystallographic information

### Crystal data

**Table:**

C_19_H_11_N_3_·C_2_H_2_Cl_4_	*F*(000) = 912.00
*M**_r_* = 449.14	*D*_x_ = 1.577 Mg m^−^^3^
Monoclinic, *C*2/*c*	Mo *K*α radiation, λ = 0.71073 Å
Hall symbol: -C 2yc	Cell parameters from 94 reflections
*a* = 20.072 (7) Å	θ = 5.4–26.8°
*b* = 14.190 (5) Å	µ = 0.64 mm^−^^1^
*c* = 7.079 (3) Å	*T* = 90 K
β = 110.255 (4)°	Needle, colorless
*V* = 1891.5 (11) Å^3^	0.79 × 0.40 × 0.10 mm
*Z* = 4	

### Data collection

**Table:**

Bruker APEXII CCD area-detector diffractometer	1606 reflections with *F*^2^ > 2σ(*F*^2^)
ω scans	*R*_int_ = 0.038
Absorption correction: multi-scan (*SADABS*; Bruker, 1996)	θ_max_ = 25.0°
*T*_min_ = 0.511, *T*_max_ = 0.938	*h* = −23→19
4336 measured reflections	*k* = −13→16
1670 independent reflections	*l* = −7→8

### Refinement

**Table:**

Refinement on *F*^2^	Secondary atom site location: difference Fourier map
*R*[*F*^2^ > 2σ(*F*^2^)] = 0.030	Hydrogen site location: inferred from neighbouring sites
*wR*(*F*^2^) = 0.081	H-atom parameters constrained
*S* = 1.06	*w* = 1/[σ^2^(*F*_o_^2^) + (0.0378*P*)^2^ + 2.3451*P*] where *P* = (*F*_o_^2^ + 2*F*_c_^2^)/3
1670 reflections	(Δ/σ)_max_ = 0.001
128 parameters	Δρ_max_ = 0.50 e Å^−^^3^
0 restraints	Δρ_min_ = −0.27 e Å^−^^3^
Primary atom site location: structure-invariant direct methods	

### Special details

**Table:**

Geometry. All e.s.d.'s (except the e.s.d. in the dihedral angle between two l.s. planes) are estimated using the full covariance matrix. The cell e.s.d.'s are taken into account individually in the estimation of e.s.d.'s in distances, angles and torsion angles; correlations between e.s.d.'s in cell parameters are only used when they are defined by crystal symmetry. An approximate (isotropic) treatment of cell e.s.d.'s is used for estimating e.s.d.'s involving l.s. planes.
Refinement. Refinement was performed using all reflections. The weighted *R*-factor (*wR*) and goodness of fit (*S*) are based on *F*^2^. *R*-factor (gt) are based on *F*. The threshold expression of *F*^2^ > 2.0 σ(*F*^2^) is used only for calculating *R*-factor (gt).

### Fractional atomic coordinates and isotropic or equivalent isotropic displacement parameters (Å^2^)

**Table:**

	*x*	*y*	*z*	*U*_iso_*/*U*_eq_	
Cl1	0.41196 (2)	0.93210 (3)	0.70600 (6)	0.02318 (16)	
Cl2	0.46383 (2)	0.83298 (3)	0.42650 (6)	0.02069 (15)	
N1	0.38456 (7)	0.62220 (9)	0.53448 (19)	0.0148 (3)	
N2	0.5000	0.61449 (12)	0.7500	0.0129 (4)	
C1	0.32689 (8)	0.57917 (11)	0.4258 (2)	0.0158 (3)	
H1	0.2883	0.6168	0.3558	0.019*	
C2	0.31719 (8)	0.47862 (11)	0.4035 (2)	0.0144 (3)	
C3	0.25243 (8)	0.43881 (12)	0.2803 (2)	0.0179 (3)	
H2	0.2145	0.4778	0.2120	0.022*	
C4	0.24495 (8)	0.34260 (12)	0.2603 (3)	0.0205 (4)	
H3	0.2021	0.3164	0.1793	0.025*	
C5	0.30269 (8)	0.28419 (12)	0.3637 (2)	0.0193 (4)	
H4	0.2978	0.2191	0.3497	0.023*	
C6	0.36626 (8)	0.32177 (11)	0.4851 (2)	0.0152 (3)	
H5	0.4039	0.2820	0.5522	0.018*	
C7	0.37471 (8)	0.42004 (11)	0.5082 (2)	0.0122 (3)	
C8	0.43950 (7)	0.46557 (10)	0.6339 (2)	0.0112 (3)	
C9	0.5000	0.41722 (14)	0.7500	0.0110 (4)	
H6	0.5000	0.3517	0.7500	0.013*	
C10	0.44225 (8)	0.56624 (10)	0.6410 (2)	0.0115 (3)	
C11	0.46206 (8)	0.83406 (10)	0.6758 (2)	0.0150 (3)	
H7	0.4386	0.7764	0.6963	0.018*	

### Atomic displacement parameters (Å^2^)

**Table:**

	*U*^11^	*U*^22^	*U*^33^	*U*^12^	*U*^13^	*U*^23^
Cl1	0.0185 (2)	0.0215 (2)	0.0267 (3)	0.00797 (15)	0.00433 (19)	−0.00421 (15)
Cl2	0.0227 (2)	0.0234 (3)	0.0136 (2)	0.00366 (15)	0.00322 (19)	−0.00042 (14)
N1	0.0132 (7)	0.0162 (7)	0.0140 (7)	0.0038 (5)	0.0035 (5)	0.0018 (5)
N2	0.0126 (9)	0.0133 (9)	0.0129 (9)	0.000	0.0043 (7)	0.000
C1	0.0119 (8)	0.0202 (8)	0.0135 (8)	0.0070 (6)	0.0023 (6)	0.0039 (6)
C2	0.0102 (7)	0.0211 (8)	0.0118 (7)	0.0020 (6)	0.0039 (6)	0.0016 (6)
C3	0.0079 (7)	0.0284 (9)	0.0154 (8)	0.0033 (6)	0.0014 (6)	0.0029 (6)
C4	0.0089 (7)	0.0298 (9)	0.0186 (9)	−0.0055 (6)	−0.0005 (7)	−0.0003 (7)
C5	0.0157 (8)	0.0187 (8)	0.0204 (8)	−0.0042 (6)	0.0026 (7)	0.0004 (6)
C6	0.0103 (7)	0.0176 (8)	0.0146 (8)	−0.0002 (6)	0.0006 (6)	0.0020 (6)
C7	0.0091 (7)	0.0174 (8)	0.0103 (7)	−0.0004 (6)	0.0036 (6)	0.0009 (5)
C8	0.0092 (7)	0.0152 (8)	0.0097 (7)	−0.0001 (6)	0.0039 (6)	−0.0002 (5)
C9	0.0108 (10)	0.0104 (10)	0.0114 (10)	0.000	0.0036 (9)	0.000
C10	0.0111 (7)	0.0136 (7)	0.0104 (8)	0.0011 (5)	0.0045 (6)	0.0010 (5)
C11	0.0158 (8)	0.0130 (8)	0.0154 (8)	0.0017 (6)	0.0046 (7)	−0.0008 (6)

### Geometric parameters (Å, º)

**Table:**

Cl1—C11	1.7722 (15)	C4—H3	0.9300
Cl2—C11	1.7778 (17)	C5—C6	1.376 (2)
N1—C1	1.299 (2)	C5—H4	0.9300
N1—C10	1.3913 (19)	C6—C7	1.407 (2)
N2—C10	1.3373 (18)	C6—H5	0.9300
N2—C10^i^	1.3373 (18)	C7—C8	1.449 (2)
C1—C2	1.441 (2)	C8—C9	1.3891 (18)
C1—H1	0.9300	C8—C10	1.430 (2)
C2—C7	1.407 (2)	C9—C8^i^	1.3891 (18)
C2—C3	1.409 (2)	C9—H6	0.9300
C3—C4	1.375 (2)	C11—C11^i^	1.522 (3)
C3—H2	0.9300	C11—H7	0.9800
C4—C5	1.406 (2)		
			
C1—N1—C10	117.16 (13)	C7—C6—H5	119.8
C10—N2—C10^i^	118.40 (18)	C6—C7—C2	118.69 (14)
N1—C1—C2	126.09 (14)	C6—C7—C8	124.04 (14)
N1—C1—H1	117.0	C2—C7—C8	117.28 (14)
C2—C1—H1	117.0	C9—C8—C10	117.23 (13)
C7—C2—C3	120.14 (15)	C9—C8—C7	123.92 (14)
C7—C2—C1	118.16 (14)	C10—C8—C7	118.86 (13)
C3—C2—C1	121.70 (14)	C8^i^—C9—C8	120.80 (19)
C4—C3—C2	120.41 (15)	C8^i^—C9—H6	119.6
C4—C3—H2	119.8	C8—C9—H6	119.6
C2—C3—H2	119.8	N2—C10—N1	114.40 (14)
C3—C4—C5	119.38 (15)	N2—C10—C8	123.16 (14)
C3—C4—H3	120.3	N1—C10—C8	122.44 (13)
C5—C4—H3	120.3	C11^i^—C11—Cl1	113.02 (9)
C6—C5—C4	121.04 (15)	C11^i^—C11—Cl2	109.00 (14)
C6—C5—H4	119.5	Cl1—C11—Cl2	109.48 (8)
C4—C5—H4	119.5	C11^i^—C11—H7	108.4
C5—C6—C7	120.35 (14)	Cl1—C11—H7	108.4
C5—C6—H5	119.8	Cl2—C11—H7	108.4
			
C10—N1—C1—C2	−0.6 (2)	C6—C7—C8—C9	−1.8 (2)
N1—C1—C2—C7	0.2 (2)	C2—C7—C8—C9	178.34 (11)
N1—C1—C2—C3	−179.50 (14)	C6—C7—C8—C10	178.32 (13)
C7—C2—C3—C4	−0.3 (2)	C2—C7—C8—C10	−1.6 (2)
C1—C2—C3—C4	179.35 (15)	C10—C8—C9—C8^i^	−0.54 (9)
C2—C3—C4—C5	−0.2 (2)	C7—C8—C9—C8^i^	179.54 (15)
C3—C4—C5—C6	0.4 (3)	C10^i^—N2—C10—N1	179.30 (14)
C4—C5—C6—C7	0.0 (2)	C10^i^—N2—C10—C8	−0.61 (10)
C5—C6—C7—C2	−0.6 (2)	C1—N1—C10—N2	180.00 (12)
C5—C6—C7—C8	179.54 (14)	C1—N1—C10—C8	−0.1 (2)
C3—C2—C7—C6	0.7 (2)	C9—C8—C10—N2	1.18 (19)
C1—C2—C7—C6	−178.97 (14)	C7—C8—C10—N2	−178.90 (11)
C3—C2—C7—C8	−179.37 (13)	C9—C8—C10—N1	−178.71 (11)
C1—C2—C7—C8	0.9 (2)	C7—C8—C10—N1	1.2 (2)

Symmetry code: (i) −*x*+1, *y*, −*z*+3/2.

### Hydrogen-bond geometry (Å, º)

**Table:**

*D*—H···*A*	*D*—H	H···*A*	*D*···*A*	*D*—H···*A*
N1—H7···C11	0.98	2.53	3.372 (3)	144
N2—H7···C11	0.98	2.57	3.206 (3)	122
